# Effect of live body weight and method of synchronization on ovulation, pregnancy rate and embryo and fetal loss in buffalo heifers

**DOI:** 10.21451/1984-3143-AR2019-0009

**Published:** 2019-11-18

**Authors:** Luigi Esposito, Donato de Nicola, Anna Balestrieri, Georgios Petrovas, Francesca Licitra, Angela Salzano, Gianluca Neglia

**Affiliations:** 1 Università degli Studi di Napoli “Federico II”, Dipartimento di Medicina Veterinaria e Produzioni Animali, Napoli, Italy; 2 Istituto Zooprofilattico Sperimentale del Mezzogiorno, Portici, Napoli, Italy; 3 Istituto Zooprofilattico Sperimentale della Sicilia, Ragusa, Italy

**Keywords:** pubescent buffalo, synchronization, weight score

## Abstract

This study aimed to assess the influence of live body weight (LBW) and age on reproductive performance in buffalo heifers synchronized by different treatments. The study was carried out on 146 Mediterranean buffalo heifers (mean age 25.3±13.4 months, LBW 424±47 kg), divided into 2 homogeneous groups and synchronized by Ovsynch-TAI Program (OVS; n = 72) or double prostaglandin administered 12 days apart (PGF; n = 74). All the buffaloes were inseminated twice and follicle dimensions and ovulation rate (OR) were assessed by ultrasound 24 and 48 h post-insemination. Pregnancy was assessed on day 25, 45 and 90 post-insemination and the incidence of late embryonic (LEM) and fetal (FM) mortality were respectively recorded. Data were analyzed by ANOVA, Chi-square test and multiple logistic regression. The LBW was significantly (P<0.05) higher in inseminated animals, compared to those that did not respond to the treatments (450.0±3.2 vs. 423.2±9.6 kg in inseminated and not inseminated heifers, respectively). Total OR was similar between groups, although OR at 24 h tended to be higher (P = 0.06) in OVS (86.7 vs. 72.9% in OVS and PGF, respectively). A (P<0.01) higher LBW was observed in ovulated heifers of PGF, while no differences were recorded in OVS. LBW affected OR (odds ratio = 1,032; P<0.05) only in PGF, while no effects were recorded in OVS. Total pregnancy rate, LEM and FM were similar between groups. In conclusion, the LBW would be considered before including buffalo heifers in a synchronization program and both synchronization treatments can be useful.

## Introduction

In buffalo species the optimization of breeding techniques in Italy allowed to reduce the age at first calving from about 45 months to less than 30 months in 30 years (Bhatti et al., 2007). The onset of puberty and, consequently, the age at first calving, is deeply affected by several factors, such as management, together with genotype and climate (Campanile et al., 2009). However, one of the main aspects influencing the reaching of puberty is the live body weight (LBW; Campanile et al., 2009). An anticipation of puberty has been observed in both Egyptian (El Nouty, 1971) and Mediterranean buffaloes (Campanile et al., 2001) that showed a daily weight gain after weaning of 0.45 and 0.55 kg, respectively. A reduction of the generation interval can be obtained anticipating the age at first calving and by the utilization of artificial insemination (AI). Recently, several synchronization protocols have been developed in buffalo, doubling the efficiency of AI (25-50% pregnancy rate) over a period of approximately two decades (Zicarelli et al., 1997; Neglia et al., 2015). However, the majority of studies has been primarily performed in adult buffaloes (Neglia et al., 2016; Monteiro et al., 2018), while few trials were carried out in heifers (Carvalho et al., 2017; Neglia et al., 2018). In cyclic buffalo heifers with normal Corpus Luteum (CL) function, the response to estrus synchronization by Prostaglandin F_2α_ (PGF_2α_; Zicarelli et al., 1997) or Ovsynch-Timed Artificial Insemination (O-TAI) program (Neglia et al., 2018), is comparable to the response observed in cattle. However, at present, no information are available on the relationships between the body weight and reproductive performance in buffalo heifers treated by different synchronization protocols. Therefore, the aims of this study were: i) to assess the influence of LBW on reproductive performance in buffalo heifers; and ii) to evaluate if the synchronization by double PGF_2α_ or O-TAI program may affect pregnancy rate in buffalo heifers.

## Methods

### Animals

The investigation was carried out in accordance with EU Directive 2010/63/EU and the Animal Ethics Committee of the University of Naples, Federico II (Permit number 2013/010858). The study was carried out on 146 Italian Mediterranean buffalo heifers, previously chosen (see below), with a mean age of 25.3±13.4 months and a mean LBW of 424±47 kg, between February and March 2017. To ensure the best welfare conditions, buffalo heifers were maintained in open yards that allowed 10 m^2^ for animal and received a total daily mixed ration consisting in 8 kg of dry matter, 0.85 UFL/kg dry matter, 13% crude protein, 18% starch and 44% NDF. Twenty and ten days before the start of the trial, the animals underwent ultrasound examination with a portable Sonoace Pico (Medison, Seoul, South Korea) equipped with a 10 MHz linear transducer for trans rectal examination. Only heifers in good health, without any abnormalities of the genital tract and with the presence of a CL in at least one examination were included in the study. Furthermore, in correspondence with the second ultrasound examination, all the animals were weighed. Only the animals with a LBW > 270 kg (n = 146) were chosen. This because puberty is related to the age at first oestrus, (at about 16.5-19.0 months) and to the LBW (at around 340-360 kg) (Borghese et al., 1994).

### Estrus synchronization treatments and AI

The selected heifers were divided into two homogeneous groups, according to LBW and age. Heifers in OVS (n = 72) underwent synchronization of ovulation by O-TAI Program (Neglia et al., 2016). Heifers in PGF (n = 74) underwent synchronization by a double PGF_2α_ analogue (Dinoprost, 25 mg; Dinolytic®, Zoetis, Rome, Italy) injection on day 0 and 12, at a random day of the cycle. AI were performed by the same operator and each buffalo was inseminated twice: at 16 and 40 hours after the second injection of GnRH in OVS and at 60 and 84 hours after the last PGF_2α_ in PGF (Neglia et al., 2008). Because of the low intensity of estrous behavior in buffaloes (Ohashi, 1994), the heifers were palpated *per rectum* to assess the estrous status (tonic uterus in presence or absence of vaginal mucus discharge) and underwent ultrasound examination to record preovulatory follicle. The ovulation rate was assessed by ultrasound in each group, 24 and 48 hours after the first insemination. Frozen/thawed semen of two bulls of proven fertility was utilized in the AI program in both groups. Finally, the body condition score was assessed on the day of AI, by using a 1 to 9 scale (Wagner et al., 1988).

### Pregnancy assessment

Twenty-five days after AI, buffaloes underwent trans rectal ultrasonography to assess embryonic development and the heartbeat. Pregnancy diagnosis was confirmed on day 45 and 90 after AI: heifers pregnant on day 25 but not on day 45 were considered to have undergone late embryonic mortality. Similarly, buffaloes pregnant on day 45 but not on day 90 were considered to have undergone fetal mortality.

### Statistical analysis

Differences in ovulation rate after 24 and 48 hours after the 1^st^ AI and pregnancy between treatments were assessed by Chi-square test. Differences in age, LBW, BCS, pregnancy rate, LEM and FM between treatments were assessed by ANOVA. Two separate logistic regression models were estimated to assess the ovulation rate at different time within each treatment with respect to the LBW. Pregnancy outcome was evaluated by a logistic regression second order model using LBW and treatment as independent variables. All statistics were performed using the IBM SPSS Statistics for Windows, version 20.0” program (IBM, 2011).

## Results

Eleven animals did not respond to the synchronization treatments and were excluded from the trial. A total of 92.5% synchronization rate was recorded, without any difference between the two synchronization treatments (93.1 vs. 91.9% in OVS and PGF, respectively). LBW was (P<0.05) higher in animals that were inseminated, compared to those that did not respond to the synchronization treatment (450.0±3.2 vs. 423.2±9.6 kg in inseminated and not inseminated heifers, respectively), whereas a similar age was recorded (796.5±9.0 vs. 789.7±40.7 days in inseminated and not inseminated heifers, respectively). No differences were recorded in preovulatory follicle dimensions between OVS and PGF, neither on the day of TAI (1.27±0.02 vs. 1.32±0.03 cm, in OVS and PGF, respectively) nor at 24 h (1.49±0.06 vs. 1.50±0.04 cm, in OVS and B, respectively). Ovulated buffaloes showed a significantly (P<0.05) higher LBW compared to not-ovulated counterparts (Table 1) regardless of the treatment, and in particular in the PGF Groupand a similar total ovulation rate was recorded between OVS and PGF. However, within each treatment, only ovulated heifers of PGF showed a significantly (P<0.01) higher LBW and age compared to those not ovulated, whereas no differences were recorded in animals synchronized by O-TAI Program (Table 1). Neither the LBW nor the age affected ovulation rate at 24 and 48 hours after insemination (data not shown). However, the ovulation rate recorded in OVS at 24 h tended to be higher (P = 0.06) compared to that recorded in PGF (86.7 vs. 72.9% in OVS and PGF, respectively). The multiple logistic regression analysis showed that ovulation rate tended to be influenced by LBW, independently of the treatment (odds ratio = 1,030; P = 0.08). If only heifers in PGF were considered, ovulation rate was significantly affected by LBW (odds ratio = 1,032; P<0.05), while no effects were recorded in OVS. Similarly, ovulation rate at 24 h was significantly influenced by LBW only in PGF (odds ratio = 1,033; P<0.01), but no effects were observed in OVS. No influence of LBW was observed for the ovulation rate at 48 h. Data on pregnancy rate are reported in Table 1. If only ovulated animals were considered, LBW tended to be higher (P = 0.08) in pregnant heifers compared to not pregnant counterparts (457.2±4.2 vs. 445.7±5.3, in pregnant and not pregnant heifers, respectively), whereas a similar age was recorded. No differences were found between OVS and PGF (Table 1) in terms of pregnancy rate and a similar live body weight and age were recorded between pregnant and not pregnant heifers within each treatment. However, a significantly higher pregnancy rate was recorded in heifers ovulated after 48 h in PGF (12/16; 75%) vs OVS (3/8; 37.5%). The regression logistic analysis for pregnancy, did not assess any influence of LBW or age on pregnancy. Finally, no differences were recorded for BCS in both pregnant and not pregnant heifers in both groups (7.2±0.2 vs. 7.3±0.2, in pregnant and not pregnant heifers of OVS, respectively and 7.1±0.2 vs. 7.2±0.2, in pregnant and not pregnant heifers of PGF, respectively).

**Table 1:** Effect of live body weight (LBW) on synchronization, ovulation and pregnancy rate and on embryo and fetal loss in buffalo heifers synchronized with Ovsynch-TAI Program (OVS) and double prostaglandin (PGF).

	**OVS**		**PGF**		**Statistical Significance**
**Synchronization rate** **%** **(n)**	Yes (67)	No (5)	Yes (68)	No (6)	
**LBW**	457.1±4.4	434.0±21.2	449.4.7^a^	414.2±2.2^b^	P = 0.04
**Ovulation rate** **%** **(n)**	Yes 60 (89.6)	No 7 (10.4)	Yes 59 (86.8)	No 9 (13.2)	
**LBW**	450.8±4.5	454.0±19.2	454.4±5.0^a^	413.2±6.7^b^	P = 0.03
**Pregnancy Rate (90 d)** **%** **(n)**	Yes 47.8 (32)	No 52.2 (35)	Yes 57.4 (39)	No 42.6 (29)	
**LBW**	456.3±5.7	446.3±6.6	458.0±6.1	436.8±6.9	
**Embryo Loss** **%** **(n)**	Yes 15.0 (6)	No 85.0 (34)	Yes 7.1 (3)	No 92.9 (39)	
**LBW**	427.8±12.5	455.1±5.5	453.3±6.9	458.0±6.1	
**Fetal Loss** **%** **(n)**	Yes 5.9 (2)	No 94.1 (32)	Yes 0.0 (0)	No 100.0 (39)	
**LBW**	434.5±13.5	456.3±5.7		458.0±6.1	
**Total embryo and fetal loss** **%** **(n)**	Yes 20.9 (8)	No 79.1 (32)	Yes 7.1 (3)	No 92.9 (39)	
**LBW**	429.5±9.6	456.3±5.7	453.3±6.9	458.0±6.1	

## Discussion

This study aimed to assess the influence of LBW on reproductive efficiency in buffalo heifers. An optimal management in this category of subjects influences the age at first calving and allows to anticipate the start of their productive life, reducing the generational interval and favoring the genetic improvement of the herd. Accordingly, reproductive development in heifers and the attainment of puberty are determined primarily by nutrition from the time of weaning (Campanile et al., 2001). Some studies performed in buffalo (Campanile et al., 2001, 2009), demonstrated that post-weaning weight gain may influence reproductive performance in heifers and the LBW on the day of insemination further affects reproduction, particularly when different protocols of synchronization are applied. In our study, LBW significantly influenced either the response to the synchronization treatment and the ovulation rate. In Mediterranean Italian buffaloes, different studies showed an age at first oestrus, stated as the first blood P_4_ rise at a value over 1 ng/ml, at about 16.5-19.0 months of age and at around 340-360 kg of body weight (Borghese et al., 1994). Cyclic ovarian activity, with a P_4_ value > 1 ng/ml at about a ten-day interval was achieved at 20.7 months of age and at a body weight of 380-390 kg (Borghese et al., 1994). On these bases, in our study all the heifers with LBW lower than 370 kg were excluded from the trial. It is worth pointing out that the influence of LBW was only observed in PGF. Synchronization by double prostaglandin needs a regular cyclic activity of the animals, as demonstrated by the evidence that anoestrus or prepubertal subjects are not responsive (Stevenson and Pursley, 1994). After the onset of puberty, LH decreased at a level similar to that of 4 months before puberty and a positive correlation between LH and body weight was found during the prepubertal period (Haldar and Prakash, 2006). On the contrary, the LBW did not affect the ovulation rate in animals synchronized by O-TAI Program. The latter is largely applied in both buffalo (Neglia et al., 2016; Sharma et al., 2017), and bovine species (Pursley et al., 1995; Carvalho et al., 2015), because of its feasibility and practical and economic advantages. The ovulation that occurs following exogenous GnRH administration is due to the presence of a follicle with ovulatory capability. In cattle, a follicle requires on average 7 to 10 days to go through the stages of emergency, deviance and dominance and then reaches the preovulatory stage or encounters atresia (Ginther et al., 1989). In buffalo the deviation phase, and hence the acquisition of LH receptors, is reached at a mean diameter of 0.75 cm (Baruselli et al., 2001). The ovulation rate recorded at 24 h tended to be higher in heifers of OVS compared to those of PGF. It is known that the last GnRH of the O-TAI treatment aims to induce an endogenous LH surge that is responsible for the synchronization of ovulation (Pursley et al., 1995). On the contrary, treatment by double prostaglandin is based on CL regression, a consequent decrease of circulating progesterone that in turn causes an increase of GnRH and LH pulse frequency, that can act on the preovulatory follicle. Therefore, in this case the ovulation is not induced and it is dependent from the maturation and the oestradiol levels of the preovulatory follicle (Kastelic and Ginther, 1991). In a previous trial carried out in adult buffaloes synchronized by O-TAI Program, it was demonstrated that the LH surge occurs about 1 hour after GnRH injection (Campanile et al., 2008). On the other hand, with the double PGF2α protocol LH surge occurs naturally and GnRH peak and hence the ovulation may be slightly delayed. In fact, in heifers ovulated at 48 h a significantly higher pregnancy rate was recorded compared to Ovsynch-treated buffaloes. In any case, no differences were observed between the two synchronization protocols in terms of pregnancy rate, suggesting that both protocols can be used in buffalo heifers. The treatment by double prostaglandin resulted in a 10% more pregnancy rate, despite of the low number of ovulated buffaloes at 24 h and the requirement of two AI. An improvement in this protocol may be obtained by administrating a GnRH two days later the last prostaglandin in order to synchronize the ovulation rate at 24 h. On the contrary, the O-TAI Program is characterized by a short duration and by the possibility of reducing the number of inseminations allowing economic advantages. In any case, a key point is the selection of the heifers, that would reach a reasonable weight and ovarian cyclicity.
